# Explaining variation in sweat sodium concentration: effect of individual characteristics and exercise, environmental, and dietary factors

**DOI:** 10.1152/japplphysiol.00391.2022

**Published:** 2022-10-13

**Authors:** Lindsay B. Baker, Peter John D. De Chavez, Ryan P. Nuccio, Shyretha D. Brown, Michelle A. King, Bridget C. Sopeña, Kelly A. Barnes

**Affiliations:** ^1^Gatorade Sports Science Institute, PepsiCo R&D, Barrington, Illinois; ^2^PepsiCo Data Science and Analytics, PepsiCo R&D, Barrington, Illinois

**Keywords:** dietary sodium, energy expenditure, ethnicity, hypohydration, race

## Abstract

This study determined the relative importance of several individual characteristics and dietary, environmental, and exercise factors in determining sweat [Na^+^] during exercise. Data from 1944 sweat tests were compiled for a retrospective analysis. Stepwise multiple regression (*P* < 0.05 threshold for inclusion) and *T* values were used to express the relative importance of each factor in a model. Three separate models were developed based on available independent variables: *model 1* (1,944 sweat tests from 1,304 subjects); *model 2* (subset with energy expenditure: 1,003 sweat tests from 607 subjects); *model 3* (subset with energy expenditure, dietary sodium, and V̇o_2max_: *n* = 48). Whole body sweat [Na^+^] was predicted from forearm sweat patches in *models 1 and 2* and directly measured using whole body washdown in *model 3*. There were no significant effects of age group, race/ethnicity, relative humidity, exercise duration, pre-exercise urine specific gravity, exercise fluid balance, or dietary or exercise sodium intake on any model. Significant predictors in *model 1* (adjusted *r*^2^ = 0.17, *P* < 0.001) were season of the year (warm, *T* = −6.8), exercise mode (cycling, *T* = 6.8), sex (male, *T* = 4.9), whole body sweating rate (*T* = 4.5), and body mass (*T* = −3.0). Significant predictors in *model 2* (adjusted *r*^2^ = 0.19, *P* < 0.001) were season of the year (warm, *T* = −5.2), energy expenditure (*T* = 4.7), exercise mode (cycling, *T* = 3.6), air temperature (*T* = 3.0), and sex (male, *T* = 2.7). The only significant predictor in *model 3* (*r*^2^ = 0.23, *P* < 0.001) was energy expenditure (*T* = 3.8). In summary, the models accounted for 17%–23% of the variation in whole body sweat [Na^+^] and energy expenditure and season of the year (proxy for heat acclimatization) were the most important factors.

**NEW & NOTEWORTHY** This comprehensive analysis of a large, diverse data set contributes to our overall understanding of the factors that influence whole body sweat [Na^+^]. The main finding was that energy expenditure was directly associated with whole body sweat [Na^+^], potentially via the relation between energy expenditure and whole body sweating rate (WBSR). Warmer months (proxy for heat acclimatization) were associated with lower whole body sweat [Na^+^]. Exercise mode, air temperature, and sex may also have small effects, but other variables (age group, race/ethnicity, fluid balance, sodium intake, relative V̇o_2max_) had no association with whole body sweat [Na^+^]. Taken together, the models explained 17%–23% of the variation in whole body sweat [Na^+^].

## Graphical abstract

**Figure:**
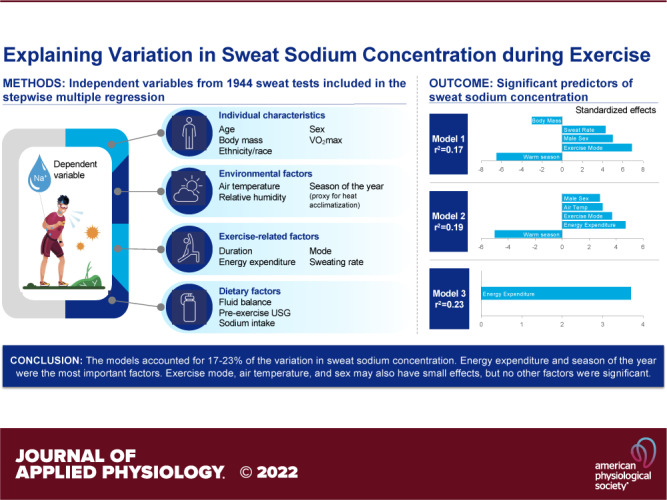

## INTRODUCTION

During exercise, the evaporation of sweat is the primary means of heat loss to counter the body heat gained as a byproduct of increased metabolism (1). In response to thermal stimuli, eccrine sweat glands are recruited to increase sweat output, leading to both water and electrolyte losses. Upon stimulation, clear cells in the secretory coil of the eccrine glands secrete primary sweat, which is nearly isotonic to the plasma in terms of sodium concentration ([Na^+^]) (2). However, final sweat is hypotonic compared with plasma [Na^+^]. This is because, in the gland duct, Na^+^ is passively reabsorbed through epithelial Na^+^ channels on the luminal membrane and actively reabsorbed through Na^+^/K^+^-ATPase transporters on the basolateral membrane (3, 4). However, because the rate of Na^+^ reabsorption in the eccrine duct varies, there is significant intra- and interindividual variation in final sweat [Na^+^] collected on the skin surface (5).

Several factors may influence the rate of Na^+^ reabsorption and, in turn, final sweat [Na^+^]. For instance, Na^+^/K^+^-ATPase activity is mediated in part by the hormonal control of aldosterone (6), which can be influenced by heat acclimation and dietary Na^+^ intake. Another factor that is known to impact final sweat [Na^+^] is sweating rate (2). As sweating rate increases with increases in absolute intensity or energy expenditure, the rate of Na^+^ secretion in precursor sweat increases proportionally more than the rate of Na^+^ reabsorption along the duct (7). Thus, there is a direct relation among energy expenditure, sweating rate, and final sweat [Na^+^] (7, 8).

Additional factors, such as sex (9), age (5, 10), race/ethnicity (11, 12), aerobic fitness (13), hydration status (14), and environmental conditions (15) have been studied to determine their potential contribution to the variation in sweat [Na^+^]. However, the few studies that have been conducted usually isolated the effect of only one independent variable on sweat [Na^+^]. In addition, differences in sweat testing methodology (e.g., regional site and collection method) can make it difficult to compare results across studies or deduce the relative importance of each contributing factor. For practical interpretation, it would be useful to conduct multiple regression analyses, taking into consideration several predictors, to determine their relative contribution to variations in sweat [Na^+^]. Elucidating underlying reasons for sweat [Na^+^] variation could help inform sweat testing guidelines and educate practitioners regarding which athletes may be at a greater risk for large sweat Na^+^ losses and what circumstances warrant more than one sweat test for a given athlete. Na^+^ ingestion along with water promotes whole body and extracellular fluid retention during/after exercise, helps maintain fluid/electrolyte balance (16), and may aid performance during prolonged exercise/heat stress that elicits large sweat Na^+^ losses (17).

Therefore, the purpose of this study was to determine the relative importance of several individual characteristics and dietary, environmental, and exercise factors in determining whole body sweat [Na^+^] during exercise. To this end a multiple regression analysis of a large data set, collected using a standardized sweat testing method, was conducted retrospectively. Based on previous literature, it was hypothesized that several independent variables (e.g., energy expenditure, air temperature, sweating rate, heat acclimatization, dietary Na^+^, and hydration status) would contribute significantly to the variation in sweat [Na^+^], with the most important factors being energy expenditure and/or sweating rate as well as heat acclimatization status.

## METHODS

### Participants

Data from 1,944 sweat tests (*n* = 1,304 subjects) were compiled from the Gatorade Sports Science Institute’s clinical research and observational service testing (collected from 2000 to 2020 in the United States) for a retrospective analysis. The data set includes 39 team/group service testing sessions, 105 individual service tests, and 13 research studies. Subject characteristics are shown in Table 1. Participants ranged from nontrained recreational exercisers to elite athletes in individual or team sports, including cycling (*n* = 270), American football (*n* = 199), basketball (*n* = 199), soccer (*n* = 174), baseball/softball (*n* = 133), running (*n* = 106), Australian rules football (*n* = 59), tennis (*n* = 40), lacrosse (*n* = 40), fitness (*n* = 36), track and field (*n* = 27), and volleyball (*n* = 21). Testing was conducted in a wide range of environmental conditions (4 to 36°C) in the laboratory or field. Of the 1,304 participants, 1,027 were male and 277 were female, while 993 were adults (≥18 yr of age) and 311 were youth (<18 yr of age). The mean ± standard deviation (SD) age was 26 ± 9 yr and body mass was 82.3 ± 20.5 kg. As this is a retrospective analysis, some of the data have been previously published to address separate research questions (18–27). All data collection received approval from an Institutional Review Board for the protection of human participants and has therefore been performed in accordance with the ethical standards in the Declaration of Helsinki. Each participant and his/her parent/guardian (for participants <18 yr) were advised of the experimental procedures and associated risks before giving their written informed consent.

**Table 1.** Descriptive data for the entire data set (1,944 sweat tests from 1,304 subjects)

	Mean	SD	Median	Range (Min to Max)
Dependent variable				
Whole body sweat sodium concentration, mmol/L	42.0	12.6	40.3	17–106
Independent variables				
Body mass, kg	82.34	20.51	78.67	38.50–164.20
Age, yr	26	9	25	12–70
Youth	16	1	16	12–17
Adult	29	8	28	18–70
Air temperature, °C	26.3	5.2	27.7	4.0–36.6
Relative humidity, %	56	15	55	13–87
Exercise duration, h	1.7	0.8	1.5	0.4–4.1
Exercise fluid balance (% change in body mass from baseline)	−0.80	0.85	−0.72	−4.38 to 2.86
Energy expenditure, kcal/h*	542	244	537	82–1735
Whole body sweating rate, L/h	1.09	0.50	1.03	0.19–5.64

* *Models 2* and *3* only.

### Experimental Design

**Figure 1.**
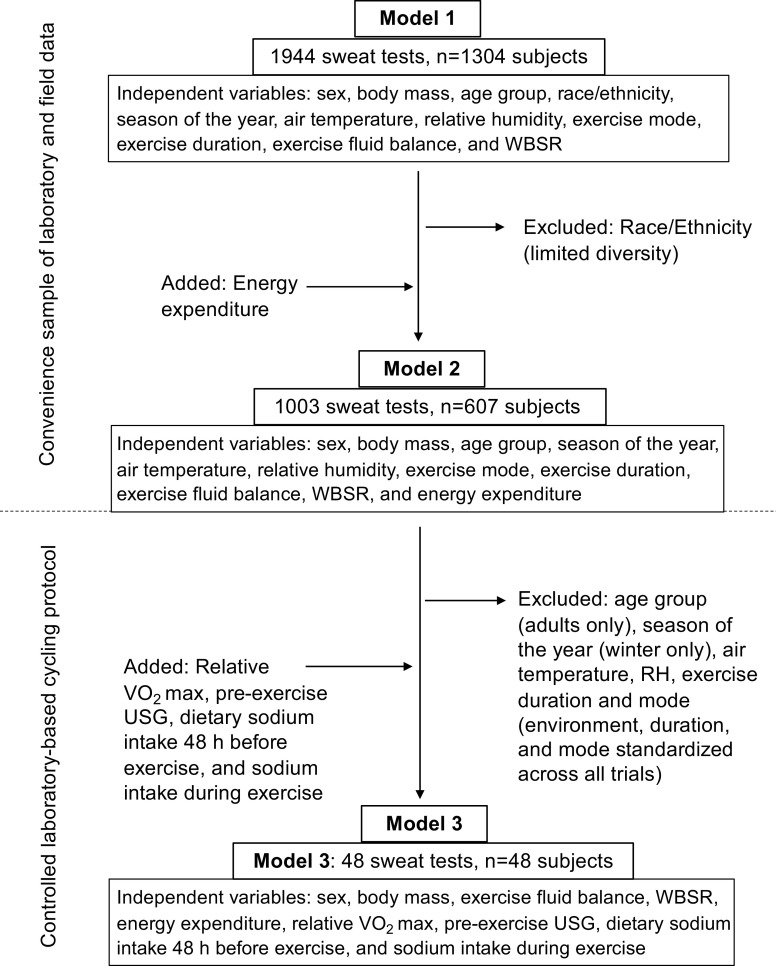
Flow chart showing independent variables included in each model and reasons for additions and exclusions in parentheses. RH, relative humidity; USG, urine specific gravity; V̇o_2max_, maximal oxygen uptake; WBSR, whole body sweating rate.

Possible independent variables for this retrospective analysis included: sex, body mass, age group (youth or adult), race/ethnicity (White/non-Hispanic, Black/non-Hispanic, Other), season of the year (cool or warm months, used as an approximation of heat acclimatization status), air temperature, relative humidity, exercise mode (type of sport/activity), exercise duration, exercise fluid balance (see *Measurements* for details), whole body sweating rate (WBSR), energy expenditure, aerobic fitness (relative V̇o_2max_), pre-exercise urine specific gravity (USG, as a measure of pre-exercise hydration status), dietary Na^+^ intake in the 48 h before exercise, and Na^+^ intake during exercise. However, certain measurements were not conducted with every sweat test. Specifically, due to practical limitations (especially with field testing), energy expenditure, V̇o_2max_, and dietary Na^+^ intake were only measured in a subset of the 1,944 sweat tests. Therefore, three separate models were developed based on the variables available (Fig. 1):

1)  *Model 1* (1,944 sweat tests, *n* = 1,304 subjects): Independent variables included sex, body mass, age group, race/ethnicity, season of the year, air temperature, relative humidity, exercise mode, exercise duration, exercise fluid balance, and WBSR.
2)  *Model 2* (1,003 sweat tests, *n* = 607 subjects): In addition to the independent variables listed for *model 1*, an estimate of energy expenditure (calculated from work rate on cycle ergometer or treadmill in laboratory trials or measured via a global positioning system (GPS)/accelerometer device in the field) was included in this subset. Race/ethnicity was removed from the analyses since there was limited diversity (75% White/non-Hispanic, 6% Black/non-Hispanic, 19% Other).
3)  *Model 3* (48 sweat tests, *n* = 48 subjects): This subset is from a controlled laboratory-based cycling protocol and included the independent variables relative V̇o_2max_, pre-exercise USG, dietary Na^+^ intake in the 48 h before exercise, and Na^+^ intake during exercise, as well as sex, body mass, exercise fluid balance, WBSR, and energy expenditure. No other variables were included because of limited diversity in age and race/ethnicity and because all sweat tests were the same exercise mode/duration (90-min cycling), completed in the same season (cool months of the year), and same air temperature (32°C).

Separate models allowed for a comprehensive analysis of all the potential independent variables in the data set. *Model 1* (full data set) was the most diverse (61% White/non-Hispanic, 20% Black/non-Hispanic, 19% other) and thus suitable for assessing the role of race/ethnicity (among other factors) on sweat [Na^+^]. The data in *model 2* was less diverse but included energy expenditure, which is expected to play an important role in determining the sweating rate, and, in turn, sweat [Na^+^]. Finally, *model 3* was used to determine the role of additional variables such as V̇o_2max_ and dietary Na^+^ intake, as well as energy expenditure and other factors, on sweat [Na^+^].

### Measurements

#### Sweat collection and analysis.

Sweat was collected from the ventral or dorsal forearm using the standard absorbent patch technique. The forearm was used because it is one of the more accessible anatomical sites, is less susceptible to patch adhesion issues than other sites, and has been shown to be a reliable predictor of whole body sweat [Na^+^] (8, 26). Before patch application, the forearm was cleaned with alcohol and/or electrolyte-free distilled or deionized water and dried with clean gauze or paper towel. The patches consisted of an absorbent pad surrounded by an occlusive adhesive dressing (Tegaderm + Pad, 3M, St. Paul, MN or Osteopatch, Pacific Biometrics, Inc. Irvine, CA). Patches were removed from the skin using clean forceps and placed in an airtight plastic tube (Sarstedt Salivette separation filter or Microsep II MF, Pall Corp., East Hill, NJ). For laboratory and most field testing, samples were extracted from the absorbent pad via centrifugation. During some field testing (*n* = 147), the sweat was squeezed out of the patch using a syringe method and analyzed on site as described in a previous study (22). Otherwise, field samples were shipped or personally transported back to the laboratory for sweat extraction and analysis within ∼1 wk of collection. When samples could not be analyzed on the day of testing, they were sealed and refrigerated at 4–8°C.

The [Na^+^] of sweat samples was analyzed using one of three possible methods: ion chromatography (Dionex ICS-3000 and ICS-6000, Thermo Fisher Scientific), flame photometry (Instrumentation Labs, IL 943, Bedford, MA), or ion-selective electrode analysis (Laqua Twin, Horiba Scientific, Edison, NJ). Samples analyzed via flame photometry and ion-selective electrode were normalized to ion chromatography using regression equations from published research (5, 22). For *models 1* and *2*, published prediction equations were applied to forearm sweat [Na^+^] to express data as whole body sweat [Na^+^] (26). For *model 3*, whole body sweat [Na^+^] was directly measured via the whole body washdown method described previously (19). Hereafter, all values reported in this paper for sweat [Na^+^] refer to whole body sweat [Na^+^].

#### Environmental conditions and season on the year.

Air temperature and relative humidity were measured during each test (QUESTemp 36, Quest Technologies, Oconomowoc, WI; Kestrel 4600 or 5400, Nielsen-Kellerman, Boothwyn, PA). The heat acclimatization status of the participants was not controlled or measured; however, the season of the year was recorded as a proxy for environmental heat exposure. The warm season was defined as testing in June through September in the northern US (mostly Illinois) or April to December in the southern US (mostly Arizona, Florida, and Texas) and may be associated with a greater potential of participants being heat acclimatized.

#### Body mass, exercise fluid balance, and sweating rate.

Before exercise, athletes were asked to void their bladder/bowels and then their body mass was measured on a digital platform scale and recorded to the nearest 0.001 kg in the laboratory (Mettler Toledo IND-690 & ICS-439) or the nearest 0.01 kg (Rice Lake IQ + 355 and Sartorius Combics 2 CISL2-U), 0.05 kg (Tanita BC-350), or 0.1 kg (Detecto DR400-750) in the field. In the laboratory nude body mass was measured (in privacy), whereas most subjects tested in the field wore minimal clothing (e.g., compression shorts/sports bra) when weighed. Upon completion of exercise, participants were asked to towel dry and were weighed using the same scale and wearing no clothing (laboratory) or the same minimal clothing (field) as the pre-exercise measurement.

To determine fluid and food intake, all drink bottles and food (and wrappers/containers, when applicable) were weighed before and after consumption (in the laboratory recorded to the nearest 0.01 g; Mettler Toledo, PG6002-S; in the field to the nearest 1 g; Ohaus CS2000). When the subjects needed to relieve themselves during exercise, urine was collected using a preweighed container and later weighed (in the laboratory to the nearest 0.01 g; Mettler Toledo PM4000, in the field to the nearest 1 g; Ohaus CS2000) to determine urine loss. When an athlete needed to have a bowel movement during exercise, their body mass was measured before and after going to the bathroom to determine mass of stool loss.

Exercise fluid balance was calculated as the change in body mass from before to after exercise, expressed as a percentage of pre-exercise body mass. Although it is acknowledged that some body mass gain (food intake) and loss (stool) occurred from solid sources, the large majority of body mass change during exercise is from sweat loss and fluid intake. Thus, for simplicity we considered a negative value for fluid balance indicative of net loss in body fluid (i.e., hypohydration), and a positive value indicative of net body fluid gain (i.e., overhydration). Note that while fluid intake was ad libitum during most field testing, some of the laboratory studies involved controlled fluid intake (based on the original research objective). Therefore, as a whole, the fluid balance data are not representative of ad libitum fluid intake behaviors. Instead, these data were included as an independent variable in the analysis to determine whether fluid balance influences sweat [Na^+^].

Finally, WBSR was calculated from the difference in pre- to postexercise body mass, corrected for food/fluid intake and urine/stool loss over the duration of exercise. We used 1.0 g/mL to convert mass to volume to express WBSR as L/h.

#### Energy expenditure (models 2 and 3 only).

When sweat testing was conducted in the laboratory, energy expenditure (kcal/h) was calculated from the cycling or running work rate (28). When energy expenditure measurements were made in the field a GPS/accelerometer device was used for stop-and-go sports (STATSports APEX Team Series, Newry, Ireland) and linear sports (Garmin Forerunner 245 and Forerunner 310XT, Garmin International, Inc. Olathe, KS).

#### Maximal oxygen consumption, sodium intake, and preexercise urine specific gravity (model 3 only).

A subset (*n* = 48, 24–41 yr) of the overall data set consisted of a 90-min cycling protocol in controlled laboratory conditions (32°C), which included measurements of relative V̇o_2max_, pre-exercise USG, dietary Na^+^ intake in the 48 h before exercise, and Na^+^ intake during exercise, as well as body mass, exercise fluid balance, WBSR, and energy expenditure. All of this testing took place from December to April in Illinois (cool season). During a preliminary screening visit before the sweat test, subjects completed a graded exercise test to estimate relative V̇o_2max_ (MOXUS; AEI Technologies, Pittsburgh, PA) on a treadmill (HPCosmos, Cosmed T200) or a cycle ergometer (Velotron SRAM, Pro). Subjects were instructed to consume their normal diet before their trials. Upon arriving at the laboratory, subjects turned in a diet log, which included specific portion sizes and brand/type for all foods, fluids, and dietary supplements consumed in the previous 48 h. The investigators reviewed the diet logs for completeness with the subjects. Na^+^ intake was determined by Registered Dietitians using the dietary analysis tool Nutribase (CyberSoft, Inc.). Na^+^ intake during exercise was determined from the volume of carbohydrate-electrolyte solution (36 mmol Na^+^/L) consumed ad libitum. A urine sample was collected upon arrival at the laboratory for assessment of baseline USG (Atago Pen Refractometer, 3741-E03, Tokyo Japan).

### Data Compilation and Quality Control Procedures

Sweat testing data for this retrospective analysis were compiled from paper and electronic files on record in our laboratory. All sweat tests with a minimum exercise duration of 0.4 h were considered for inclusion and were scrutinized for validity. Sweat [Na^+^] data were excluded under the following conditions: the athlete had a known medical condition that affects sweat [Na^+^] (i.e., cystic fibrosis); sweat [Na^+^] was measured from any site other than the ventral or dorsal forearm or with a method other than the standard regional absorbent patch technique (e.g., arm bag); the sweat patch became detached from the subject’s arm during testing, or the corresponding sweat potassium concentration was above normal physiological range (>10 mmol/L) indicative of possible sample evaporation or contamination (29).

### Statistical Analyses

Data were analyzed using Minitab 19 Statistical Software (Minitab Inc. State College, PA). Data are expressed as means ± SD, unless indicated otherwise. A stepwise regression analysis was used to determine the factors explaining the most variance in sweat [Na^+^], with *P* < 0.05 used as the threshold for inclusion in the models. Q-Q plots and Shapiro–Wilk tests were used to assess the normality of the residuals. If the normality assumption was not satisfied, the Box-Cox transformation technique was used to identify the optimal data transformation. Multicollinearity was assessed using the variance inflation factor statistic and a value of <10 was deemed acceptable. *T* values calculated from coded regression coefficients and Pareto analyses were used to express the relative importance of each factor in a model.

## RESULTS

Descriptive data for sweat [Na^+^], body mass, age group, environmental conditions, exercise duration, exercise fluid balance, energy expenditure, and WBSR for the entire data set are shown in Table 1. The sample sizes for each category of sex, age group, race/ethnicity, and season of the year for *models 1* and *2* are shown in Table 2. Descriptive data for sweat [Na^+^] and all independent variables included in *model 3* are shown in Table 3.

**Table 2.** Sample size for each category of sex, age, race/ethnicity, and season of the year

	*Model 1*	*Model 2*
Sweat tests	1,944	1,003
Subjects	1,304	607
Sex		
Male	1,027	416
Female	277	191
Age group		
Adults (≥18 yr)	993	430
Youth (<18 yr)	311	177
Race/ethnicity		
White/non-Hispanic or Latino	789	
Black/non-Hispanic or Latino	266	
Other race/ethnicity	249	
Season of the year		
Warm*	868	432
Cool	436	175

*Testing in June through September in the Northern US (mostly Illinois) or April to December in the Southern US (mostly Texas, Arizona, and Florida) was considered the “warm” season and may be associated with a greater potential of participants being heat acclimatized. Race/ethnicity not included as an independent variable in *model 2* because of limited diversity (*n* = 435 White/non-Hispanic or Latino, *n* = 35 Black/non-Hispanic or Latino, *n* = 119 Other).

**Table 3.** Variables included in laboratory-based cycling subset (model 3)

	Mean	SD	Median	Range (Min to Max)
Dependent variable				
Measured whole body sweat [Na^+^], mmol/L	41.4	15.6	39.2	15.4–92.8
Independent variables				
Pre-exercise body mass, kg	74.75	12.34	72.16	55.03–100.26
Relative maximal oxygen consumption, mL/kg/min	48.4	8.1	47.3	31.6–65.6
Exercise energy expenditure, kcal/h	639	150	629	425–976
Fluid balance (% change in body mass from baseline)	−0.99	0.71	−1.03	−2.21 to 1.34
Pre-exercise urine specific gravity	1.010	0.008	1.007	1.002–1.027
Whole body sweating rate, L/h	0.94	0.32	0.91	0.43–1.86
Exercise sodium intake, mmol	32	18	27	9–100
Dietary sodium intake 48-h before exercise, mmol/day	176	80	162	75–402

*n* = 48 (33 men, 15 women; 24–41 yrs; 43 White/non-Hispanic; 2 White/Hispanic, 2 Asian/non-Hispanic, 1 Black/non-Hispanic).

**Figure 2.**
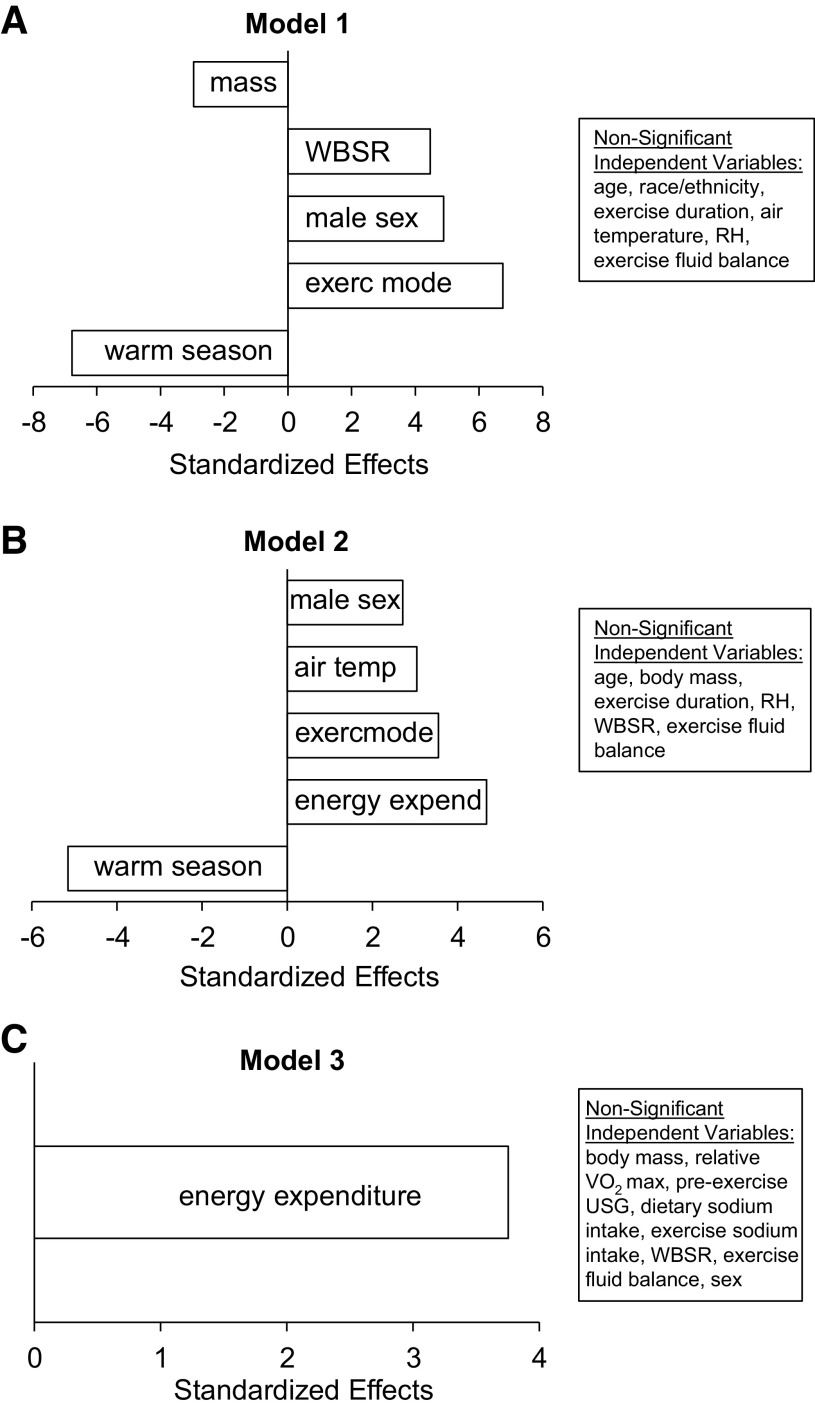
Pareto chart from the stepwise multiple regression *model 1* (*A*), *model 2* (*B*), and model 3 (*C*). Only the statistically significant independent variables are shown in the figures. RH, relative humidity; USG, urine specific gravity; V̇o_2max_, maximal oxygen uptake; WBSR, whole body sweating rate.

Table 4 shows the stepwise multiple regression results for *models 1*, *2*, and *3*. Significant predictors in *model 1* (adjusted *r*^2^ = 0.17, *P* < 0.001) were season of the year (warm, *T* = −6.8), exercise mode (cycling, *T* = 6.8), sex (male, *T* = 4.9), WBSR (*T* = 4.5), and body mass (*T* = −3.0). Significant predictors in *model 2* (adjusted *r*^2^ = 0.19, *P* < 0.001) were season of the year (warm, *T* = −5.2), energy expenditure (*T* = 4.7), exercise mode (cycling, *T* = 3.6), air temperature (*T* = 3.0), and sex (male, *T* = 2.7). The only significant predictor in *model 3* (*r*^2^ = 0.23, *P* < 0.001) was energy expenditure (*T* = 3.8). There were no significant effects of age group, race/ethnicity, relative humidity, exercise duration, pre-exercise USG, exercise fluid balance, or dietary or exercise Na^+^ intake on any of the models explaining the variance in sweat [Na^+^]. The Pareto chart with standardized effects of the significant factors in each model is shown in Fig. 2.

**Table 4.** Stepwise multiple regression results

	*r* ^2^	Independent Variable	VIF	*T* Value	*P* Value
*Model 1*	0.17 (*P* < 0.001)	Season (warm)	1.3	−6.8	*<0.001*
		Exercise mode (cycling)*	1.1–2.2	6.8	*<0.001*
		Sex (male)	1.4	4.9	*<0.001*
		WBSR	1.6	4.5	*<0.001*
		Body mass	2.5	−3.0	*0.003*
*Model 2*	0.19 (*P* < 0.001)	Season (warm)	1.2	−5.2	*<0.001*
		Energy expenditure	2.3	4.7	*<0.001*
		Exercise mode (cycling)#	1.1–2.3	3.6	*<0.001*
		Air temperature	2.6	3.0	*0.002*
		Sex (male)	1.3	2.7	*0.007*
*Model 3*	0.23 (*P* < 0.001)	Energy expenditure	1.0	3.8	*<0.001*

*r*^2^ values shown are the adjusted *r*^2^ for *models 1* and *2* and nonadjusted for *model 3*. Reference terms for independent variables are shown in parentheses. VIF, variance inflation factor; WBSR, whole body sweating rate.

* Significant modes in *model 1* were AFL (*T* = −6.0), American football (*T* = −3.2), baseball (*T* = −9.0), basketball (*T* = −6.9), lacrosse (*T* = −2.6), running (*T* = 2.7), and soccer (*T* = −7.8); #significant modes in *model 2* were baseball (*T* = −2.8), fitness (*T* = 2.6), and running (*T* = 2.3).

**Figure 3.**
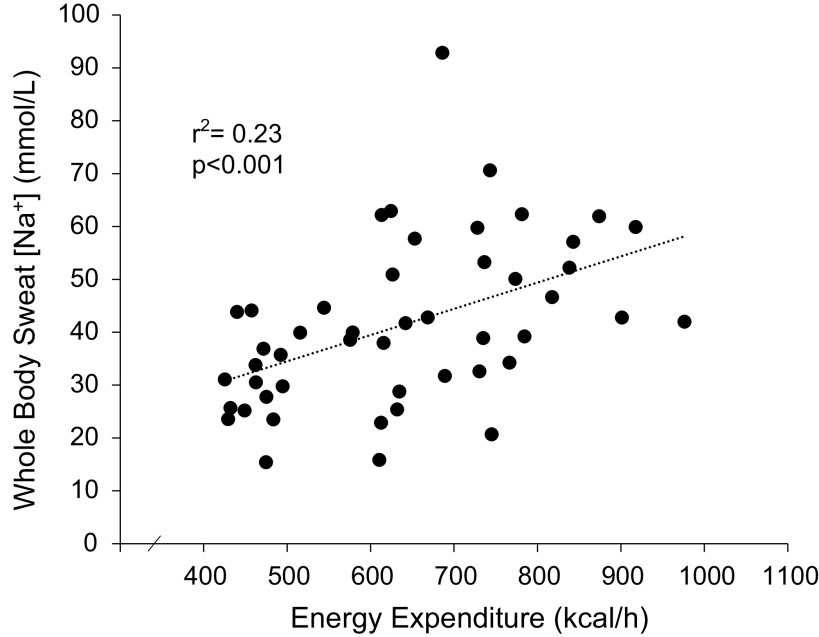
Simple linear regression of whole body sweat sodium concentration plotted as a function of energy expenditure during 90 min of cycling exercise in the heat.

The correlation matrix in Table 5 shows high correlations among body mass, WBSR, relative V̇o_2max_, energy expenditure, and/or sweat [Na^+^] for *model 3* data set. However, the multiple regression analysis revealed that energy expenditure was the only factor contributing significantly to the variation in sweat [Na^+^]. That is, energy expenditure had more impact on sweat [Na^+^] in the presence of other predictors and therefore was retained while WBSR and other factors were removed from the model by stepwise regression. To illustrate individual data points, Fig. 3 shows the simple linear regression of sweat [Na^+^] plotted as a function of energy expenditure.

**Table 5.** Correlation matrix for model 3

Pearson Correlation Coefficients *P* Values									
	Body Mass	V̇o_2max_	Energy Expenditure	Fluid Balance	Pre-Exercise USG	WBSR	Exercise Sodium Intake	Dietary Sodium Intake 48 h Before Exercise	WB Sweat Sodium Concentration
Body mass	1								
V̇o_2max_	0.08	1							
	*0.585*								
Energy expenditure	0.68	0.59	1						
	*<0.0001*	*<0.0001*							
Fluid balance	−0.26	−0.23	−0.45	1					
	*0.073*	*0.123*	*0.002*						
Pre-exercise USG	0.38	0.08	0.20	−0.31	1				
	*0.008*	*0.571*	*0.176*	*0.032*					
WBSR	0.74	0.45	0.87	−0.53	0.39	1			
	*<0.0001*	*0.001*	*<0.0001*	*0.0001*	*0.006*				
Exercise sodium intake	0.25	0.28	0.31	0.62	−0.05	0.31	1		
	*0.083*	*0.056*	*0.030*	*<0.0001*	*0.760*	*0.031*			
Dietary sodium intake 48 h before exercise	0.20	0.30	0.32	−0.13	0.10	0.25	0.13	1	
	*0.182*	*0.037*	*0.028*	*0.392*	*0.488*	*0.081*	*0.377*		
WB sweat sodium concentration	0.37	0.11	0.48	−0.28	0.28	0.41	0.05	−0.008	1
	*0.009*	*0.440*	*0.0006*	*0.053*	*0.054*	*0.004*	*0.751*	*0.959*	

USG, urine specific gravity; V̇o_2max_, maximal oxygen uptake; WB, whole body; WBSR, whole body sweating rate. Italicized numbers are *P* values.

## DISCUSSION

This analysis of a large, diverse population of athletes contributes to our overall understanding of factors that influence sweat [Na^+^]. The findings suggest that there are multiple factors impacting variation in whole body sweat [Na^+^]. Specifically, higher energy expenditure was associated with higher sweat [Na^+^], which may be explained in turn by the relation between energy expenditure and WBSR. On the other hand, heat acclimatization (estimated from season of the year) was associated with lower sweat [Na^+^]. Air temperature, exercise mode, and sex may also have small effects, but other variables included in this analysis (age group, race/ethnicity, fluid balance, Na^+^ intake, relative V̇o_2max_) had no association with sweat [Na^+^]. Overall, 17%–23% of the variation in whole body sweat [Na^+^] was explained in the multiple regression models. By comparison, previous studies have found that regional sweat [Na^+^] via the standard absorbent patch method explains ∼60%–75% of the variation in whole body sweat [Na^+^] (26). Taken together this suggests that it would be difficult to predict sweat [Na^+^] without individualized sweat patch testing. Furthermore, multiple tests within athletes may be needed for training/competitions that elicit significantly different sweating rates (e.g., exercise intensity or air temperature), involve a different mode of exercise (e.g., cross-training or multisport athletes), or are performed during different seasons of the year.

One of the most important factors in the multiple regression models was season of the year, which was used as a proxy for heat acclimatization. There was an inverse relation between season of the year and sweat [Na^+^], such that tests in warm months were associated with lower sweat [Na^+^]. It is well accepted that heat acclimation can lead to conservation of Na^+^ related to alterations in the hormonal control of Na^+^ reabsorption by aldosterone (30). Sweat [Na^+^] starts to decrease after 2–3 consecutive days of heat exposure and continues over time (31, 32), resulting in an up to 30%–60% decrease after 7–10 days (30–34). Previous studies have also reported seasonal variation, amounting to a ∼30%–60% decrease in sweat [Na^+^] from winter to summer (35, 36).

Energy expenditure was another important predictor of sweat [Na^+^] included in the models in this study. Indeed, energy expenditure was the only significant factor in *model 3* and accounted for more variation (23%) than any other individual factor in *models 1*–*2*. The underlying physiological mechanism explaining these results may be related to the well-established association between sweating rate and sweat [Na^+^] (7). Higher energy expenditure results in greater metabolic heat production, thereby increasing the evaporative requirement for heat balance. Thus, higher sweating rates associated with higher energy expenditure may have led to higher sweat [Na^+^]. Several studies have reported a direct relation between sweating rate and final sweat [Na^+^] (2, 7, 37–40). This finding has held true whether measured in isolated sweat glands (2), within given skin regions, particularly on the upper body (7, 38), or via the whole body washdown technique (8). For example, one study found that an increase in WBSR concomitant with a 38% increase in absolute energy expenditure led to a 62% increase in whole body sweat [Na^+^] (8). In addition, Buono et al. (7) found that as forearm sweating rate increased from ∼0.25 to 0.82 mg/cm^2^/min (induced via exercise intensity from 50% to 90% maximal heart rate), sweat [Na^+^] increased from 19 to 59 mmol/L (210% increase).

Like energy expenditure, higher environmental heat load increases the evaporative requirement for heat balance, which may explain air temperature’s significant, albeit smaller, effect on sweat [Na^+^] (*model 2*). Previous studies have also reported an increase in sweat [Na^+^] with higher air temperatures. For example, Dziedzic et al. (15) found that sweat samples collected in hot conditions (31.9°C) produced 15% higher sweat [Na^+^] than those from the temperate environment (21.2°C). In the present study, male sex was associated with significantly higher sweat [Na^+^]. The physiological mechanism underlying the small effect of sex is unclear since previous studies suggest that biological sex does not play an independent role in sweat [Na^+^] (9). While men tend to have higher WBSR than women this is usually attributed to differences in body mass and/or energy expenditure (41).

Counter to our hypothesis, exercise mode was a significant factor contributing to sweat [Na^+^]. However, this finding may be explained by differences in energy expenditure and/or WBSR among sports. For example, cycling, which involved continuous moderate-intensity exercise, had the highest (most positive) association with sweat [Na^+^]; with a *t* value of 6.8 and 3.6 for *models 1* and *2*, respectively. By comparison, baseball, which is a very low-intensity sport, had the lowest (most negative) association with sweat [Na^+^]; with a *t* value of −9.0 and −2.8, respectively.

It is also interesting to discuss nonsignificant factors in the sweat [Na^+^] models. Several cross-sectional studies have been conducted to determine the impact of aerobic capacity on sweat [Na^+^], but results have been mixed (for review, see Ref. 42). It may be that any improvements in eccrine duct Na^+^ reabsorption elicited by aerobic training are nullified when accounting for exercise intensity and WBSR. For age group and ethnicity/race, our results are generally in line with previous studies. For example, while Meyer et al. (10) found that men (mean = 23 yr) had a higher sweat [Na^+^] than prepubertal boys (mean = 9 yr), there was no significant difference between men and pubertal boys (mean = 11 yr). And the youth in the present study were more similar in age to the pubertal group than the prepubertal group of Meyer et al. (10). In addition, in the Meyer et al. (10) study, exercise was standardized for relative intensity, thus their lower energy expenditure may explain in part the prepubertal boys’ lower sweat [Na^+^] versus men. In the present study, age was treated as a binary variable (youth vs. adult), so the effect of aging on sweat [Na^+^] was not specifically assessed. Inoue et al. (43) found no differences in sweat [Na^+^] between young adults and 60+ year old men before or after a heat acclimation regimen; however, sweating rates differed between age groups. It may be difficult to isolate the effect of age on sweat [Na^+^] unless absolute intensity and/or sweating rate are standardized. The present study attempted to address this limitation via a multiple regression analysis and the results suggest there were no differences in sweat [Na^+^] between youth (12–17 yr) and adults (18–70 yr) when accounting for energy expenditure and WBSR.

Few well-controlled studies have assessed the effect of race/ethnicity on sweat [Na^+^], but the general consensus is that there is no evidence of inherent differences (44). For example, McLean et al. (45) found no differences between New Zealand European and Maori/Pacific Island athletes during a 1-h spin cycle exercise session in which sweating rates were well matched. Another study (46) conducted sweat testing with tropical native runners during 30–100 min at a self-selected pace and reported a similar range in sweat [Na^+^] (11–80 mmol/L) as previous normative data in athletes (5, 47), but no control groups were included. The present study suggests no impact of race/ethnicity when White/non-Hispanic, Black/non-Hispanic, and Other (broad term for all other race/ethnicity) are included in the model. However, the present study did not account for indigenous climate, as a long-term adaptation to indigenous environmental factors may be more important than race or ethnicity per se in the physiological responses to heat stress. For example, heat habituation, characterized in part by lower, more efficient sweating (less dripping) may occur in people indigenous to hot or tropical climates (48). However, it is less clear what effect indigenous climate may have on sweat [Na^+^]. Future research with greater diversity and balance among racial/ethnic groups as well as consideration for indigenous climate may be warranted to corroborate our findings.

It was somewhat surprising that fluid balance and Na^+^ intake were not significant factors in the sweat [Na^+^] models. Previous research has found that acute hypohydration (2.4% body mass loss) induced via 2-h cycling in the heat without fluid replacement was associated with 12% higher sweat [Na^+^] than when subjects maintained euhydration during exercise-heat stress (14). In addition, research has consistently shown that sweat [Na^+^] is altered after dietary Na^+^ is increased or decreased significantly from usual intake for ≥3 days (for review, see Ref. 49). For example, in a randomized controlled trial subjects’ usual Na^+^ intake of 46 mg/kg/day was increased to 100 mg/kg/day or decreased to 15 mg/kg/day for 3 days in a crossover design. Sweat [Na^+^] following the usual diet was 10%–11% higher than the low Na^+^ diet and 10%–12% lower than the high Na^+^ diet (50). It is important to acknowledge, that in the present study, dietary Na^+^ intake was assessed for only 48 h before sweat testing. However, subjects were instructed to consume their typical diet during this time so that the data would be representative of their usual Na^+^ intake. At any rate, the results of the present study do not necessarily dispute the previous controlled intervention studies that found differences in sweat [Na^+^] when they manipulated fluid balance or dietary Na^+^ to extremes of high or no/low intake. However, it seems that when taking into account several variables that could impact sweat [Na^+^], the most important are energy expenditure and season of the year (heat acclimatization). This notion is supported not only by the results of the present study, but also the relative changes in sweat [Na^+^] reported in the individual studies discussed earlier.

It is important to note the potential limitations of this study, including the estimated nature of some of the variables. Heat acclimatization was crudely estimated based on season of the year in which testing was conducted. Furthermore, energy expenditure for field testing in *model 2* was measured via GPS/accelerometer-based measurements, which are generally known to have limited accuracy, especially for nonlinear sports (51). It is possible that factors correlated with EE, such as sex and exercise mode, were significant predictors of sweat [Na^+^] in *model 2* because of the limited accuracy of the field-based energy expenditure measurements. Interestingly, sex was not a significant factor in *model 3* where energy expenditure was determined from laboratory-based measurements of workload. Finally, for *models 1* and *2* whole body sweat [Na^+^] was predicted from regional measurements from the dorsal and ventral forearm. This was done to normalize the dependent variable across all trials and aid in practical interpretation. Although such indirect measurements help enable the compilation of a large data set, these limitations may explain in part why the models explained only 17%–23% of the variation in whole body sweat [Na^+^].

In conclusion, this comprehensive analysis of 1,944 sweat tests from 1,304 subjects suggests that higher energy expenditures are associated with higher sweat [Na^+^] and tests in warmer months (proxy for heat acclimatization) are associated with lower sweat [Na^+^]. Exercise mode, air temperature, and sex may also have small effects, and taken together, the models explained 17%–23% of the variation in sweat [Na^+^]. Other variables included in the analyses, such as age group, race/ethnicity, fluid balance, Na^+^ intake, and relative V̇o_2max_ were not associated with sweat [Na^+^].

## GRANTS

This study was funded by the Gatorade Sports Science Institute, a division of PepsiCo R&D.

## DISCLOSURES

Authors are employed by PepsiCo R&D. The views expressed in this manuscript are those of the authors and do not necessarily reflect the position or policy of PepsiCo, Inc.

## AUTHOR CONTRIBUTIONS

L.B.B. and K.A.B. conceived and designed research; L.B.B., P.J.D.D., R.P.N., S.D.B., M.A.K., B.C.S., and K.A.B. performed experiments; L.B.B., P.J.D.D., and R.P.N. analyzed data; L.B.B. and P.J.D.D. interpreted results of experiments; L.B.B. prepared figures; L.B.B. drafted manuscript; L.B.B., P.J.D.D., R.P.N., S.D.B., M.A.K., and K.A.B. edited and revised manuscript; L.B.B., P.J.D.D., R.P.N., S.D.B., M.A.K., B.C.S., and K.A.B. approved final version of manuscript.
